# Collaborative Molecular Epidemiology Study of Metabolic Dysregulation, DNA Methylation, and Breast Cancer Risk Among Nigerian Women: MEND Study Objectives and Design

**DOI:** 10.1200/JGO.18.00226

**Published:** 2019-06-13

**Authors:** Tomi Akinyemiju, Omolola Salako, Adetola Daramola, Olusegun Alatise, Adewale Adeniyi, Gabriel Ogun, Omobolaji Ayandipo, Thomas Olajide, Olalekan Olasehinde, Olukayode Arowolo, Adewale Adisa, Oludolapo Afuwape, Aralola Olusanya, Aderemi Adegoke, Akinlolu Ojo, Trygve Tollefsbol, Donna Arnett

**Affiliations:** ^1^ University of Kentucky, Lexington, KY; ^2^ Lagos University Teaching Hospital, Lagos, Nigeria; ^3^ Obafemi Awolowo University Teaching Hospital, Ile-Ife, Nigeria; ^4^ Federal Medical Center, Abeokuta, Nigeria; ^5^ University of Ibadan, Ibadan, Nigeria; ^6^ Our Lady of Apostle Catholic Hospital, Ibadan, Nigeria; ^7^ University of Arizona, Tucson, AZ; ^8^ University of Alabama at Birmingham, Birmingham, AL

## Abstract

**PURPOSE:**

To elucidate the role of metabolic dysregulation and associated DNA methylation changes on breast cancer risk and aggressive subtypes among Nigerian women. We describe the design and methods of a collaborative molecular epidemiology study of breast cancer in Nigerian hospitals.

**METHODS:**

The Mechanisms for Novel and Established Risk Factors for Breast Cancer in Women of Nigerian Descent (MEND) study was designed as a matched case-control study of 350 patients, age 18 to 75 years, with newly diagnosed, treatment-naïve breast cancer and 350 age-matched healthy controls from surrounding geographic areas. Patients with breast cancer seen for initial diagnosis at four large tertiary hospitals in southwest Nigeria and one affiliated private hospital were recruited. Healthy female controls were selected from a cohort of 4,000 healthy women recruited as part of the Human Heredity and Health (H3) in Africa Chronic Kidney Disease Case-Control Study in Nigeria. Tumor and adjacent normal tissue, and blood and saliva samples were collected for molecular and epigenetic assays.

**RESULTS:**

Although recruitment is ongoing, a total of 416 patients have been recruited to date, with tumor and blood samples obtained from at least 310 patients. Data on age-matched (± 6 months) controls have also been obtained and harmonized. Lipid assays for 350 pathologically verified cases and 350 age-matched controls is underway, and pathologic characterization of tumors (including immunohistochemistry for subtyping) is ongoing. Data on DNA methylation for tumors and adjacent normal tissue are expected by the end of the study period.

**CONCLUSION:**

The MEND study will provide a unique, high-quality source of data to evaluate the contribution of metabolic dysregulation such as obesity, diabetes, hypertension, and metabolic syndrome to the biology of breast cancer among Nigerian women and foster collaborative studies relevant for women of African descent globally.

## INTRODUCTION

Developing countries accounted for 45% of new breast cancer cases globally in 2009 and 57% in 2012, with these proportions projected to increase to 70% by 2030.^1,2^ This dramatic increase is consistent with the epidemiologic transition, a stage in a country’s economic development characterized by declines in infectious diseases and increases in noncommunicable diseases as the leading causes of death, coinciding with an increase in the proportion of people living above the poverty level.^3^ Nigeria, accounting for one-sixth of the entire African population, is classified by the World Bank as a lower-middle income country with a rapidly growing economy^4^ and is swiftly undergoing an epidemiologic transition. This is evidenced by an increase in the prevalence of so-called diseases of affluence that result in higher rates of metabolic dysregulation, characterized by obesity, hypertension, diabetes, and metabolic syndrome (MetS).^5^ These cardiometabolic risk factors are associated with substantially increased risk of coronary heart disease, stroke, and type-2 diabetes,^6-11^ and prevalence of MetS is estimated to be 35% to 43% in urban areas of Nigeria.^12,13^ Given a consistent association with increased risk of breast cancer in other settings,^14,15^ the coinciding epidemics of MetS and breast cancer in Nigeria are cause for concern.

In Nigeria, breast cancer incidence increased three-fold from 15 cases per 100,000 in 1973 to 52 cases per 100,000 in 2012.^16,17^ Breast cancer in Nigeria also exhibits several epidemiologic features that are striking when compared with the disease in developed countries. First, the majority of cases are premenopausal, with more than 70% of patients diagnosed between ages 20 and 50 years^16,18^; this is similar to patterns observed among women of African descent in the United States and the United Kingdom but in contrast with predominantly postmenopausal breast cancer observed among white women.^19-21^ Many established risk factors for breast cancer, such as low parity and lack of breastfeeding, are less common in Nigerian women and typically are associated with postmenopausal breast cancer^22-24^; however, risk factors for premenopausal breast cancer are not well understood. Second, 60% to 80% of patients with breast cancer in Nigeria are diagnosed at stages III or IV with high-grade disease,^25-28^ because of a combination of infrequent screening and fast-growing tumors. Comparably aggressive phenotypes have also been documented among women of African descent in the United States and the United Kingdom, where better screening strategies exist, whereas less aggressive phenotypes are observed in white women.^27,29-31^ Third, several studies have reported that, similar to women of African descent in the United States and United Kingdom, but in contrast with white women, breast tumors in Nigerian women are more likely to be receptor negative for estrogen, progesterone, and human epidermal growth factor 2 (ie, triple-negative breast cancer).^28,32^ Fourth, differential DNA methylation of genes involved in tumor suppression and DNA repair has been implicated in young onset, aggressive, triple-negative breast cancer subtypes among black women versus with white women,^33^ although this pattern has not been well characterized in Nigerian women.

**FIG 1:**
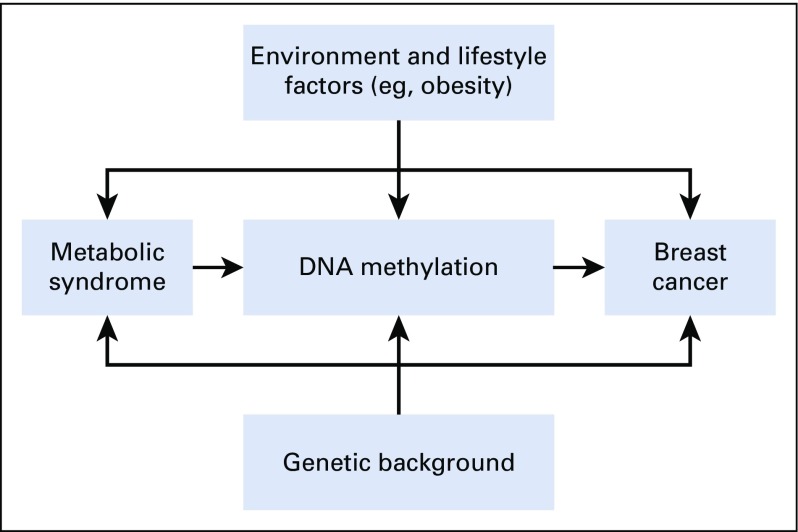
Conceptual framework of metabolic syndrome and breast cancer. Adapted from Hauner et al.^14^

Despite the striking similarities in breast cancer phenotypes among Nigerian women and other women of African descent and in contrast with white women, molecular epidemiology studies characterizing the biologic mechanisms associated with the aggressive subtypes frequently observed among women of African descent remain scarce. Metabolic dysregulation may influence breast cancer etiology, aggressiveness, and outcomes via pathways that involve (1) higher circulating insulin levels leading to mitogenic, antiapoptotic, and angiogenic properties; (2) chronic inflammation; and (3) increased visceral fat stores and other cancer-associated adipokines that may promote tumor growth.^34^ However, no study, to our knowledge, has evaluated whether DNA methylation, given its sensitivity to environmental and genetic cues, may provide additional insights into unique biologic mechanisms through which metabolic dysregulation or MetS increases risk of breast cancer and aggressive subtypes in Nigerian women (Fig 1). We hypothesize that MetS may induce differential methylation in key breast cancer–related genes, leading to increased breast cancer risk. We previously reported an association between MetS and differential DNA methylation in the *ABCG1* gene involved in cellular cholesterol transport and found to be altered in approximately 35% of breast cancer cases.^35^ The current study will provide critical epidemiologic and clinical data on a wide range of novel and established breast cancer risk factors that can serve as a basis for future investigations into determinants of breast cancer risk and survival among African women in Nigeria. Here, we describe the design and methods of the Mechanisms for Novel and Established Risk Factors for Breast Cancer in Women of Nigerian Descent (MEND) study.

### Objectives of MEND

The MEND study was designed to generate high-quality epidemiologic, molecular, and epigenetic data regarding the role of metabolic dysregulation in breast cancer risk and subtypes in Nigeria. The primary aims are as follows:

1. To recruit 350 patients (18 to 75 years of age) with newly diagnosed, treatment-naïve breast cancer from tertiary hospitals in Nigeria, and 350 age-matched healthy controls;
2. To determine the prevalence of metabolic dysregulation (ie, obesity, diabetes, high blood pressure, dyslipidemia, elevated triglyceride levels, and MetS) among cases and controls, and estimate the association between MetS and components with breast cancer subtype; and
3. To characterize genome-wide DNA methylation levels in tumor and adjacent normal tissue from incident breast cancer cases and identify differentially methylated genes by MetS status.

## METHODS

### Study Design and Organization:

The study was originally designed as a matched case-control study of 350 patients, 18 to 75 years of age, with newly diagnosed, treatment-naïve incident breast cancer, and 350 age-matched healthy controls from surrounding geographic areas (Appendix; Appendix Table A1). The organization of MEND comprises a team of collaborators from four tertiary hospitals and one private hospital in southwest Nigeria: Lagos University Teaching Hospital, Idi-Araba, Lagos State; Federal Medical Center, Idi-Aba, Abeokuta, Ogun State; University College Hospital, Ibadan, Oyo State; Our Lady of Apostle Catholic Hospital, Ibadan, Oyo State; and Obafemi Awolowo University Teaching Hospital, Ile-Ife, Osun State. The hospital sites were selected on the basis of location in densely populated regions of the country to enhance a rapid pace of recruitment; reputable tertiary hospitals with established breast cancer clinics; collaborative opportunity with breast cancer specialists to facilitate administrative approvals and patient recruitment; and proximity to commercial or urban city centers to facilitate transfer of supplies to and from Nigeria. Research teams at each site included at minimum a pathologist, oncologist (medical, radiologic, and/or surgical), research nurse, and laboratory technician. Study methods were reviewed and approved by collaborators and institutional review boards at each research site, as well as the institutional review boards at the University of Alabama at Birmingham and the University of Kentucky.

### Recruitment and Baseline Assessment

Recruitment of cases and collection of baseline data continues and were designed to occur primarily during the initial diagnostic visit, on the basis of a probable breast cancer diagnosis by an oncologist but before pathologic confirmation. This approach ensured that data, physical measurements, and samples were obtained before treatment, and reduced the impact of patient loss to follow-up, a common occurrence in this setting.

### Breast Cancer Cases

**FIG 2:**
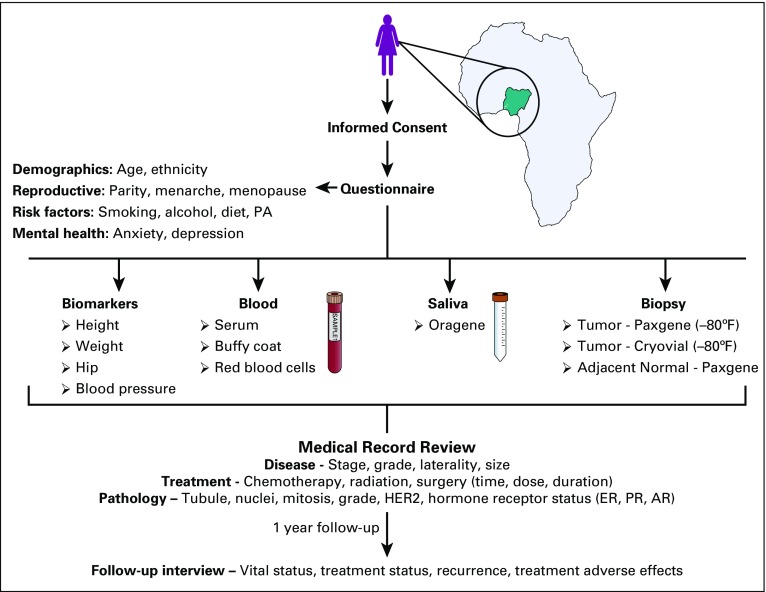
Schema of the mechanisms for novel and established risk factors for breast cancer in women of Nigerian descent study design. AR, androgen receptor; ER, estrogen receptor; HER2, human epidermal growth factor receptor 2; Oragene, Oragene Saliva Collection Kit; PA, physical activity; PR, progesterone receptor.

Figure 2 illustrates the study procedure. After a patient received an initial, probable diagnosis of breast cancer, the research study was briefly explained to the patient and if the patient expressed interest, the research nurse fully explained the study requirements in an adjoining examination room. Patients were free to refuse to participate at any point during the study. Eligibility was determined on the basis of a series of initial questions, and exclusion criteria included previous diagnosis and/or treatment of cancer, medical conditions that could prevent long-term participation, cognitive impairment judged by the research nurse, or inability to communicate in English without a family member who could translate. The research nurse then completed the informed consent procedure and obtained verbal and written informed consent. Because English is the official language of Nigeria, most patients understood and spoke at least some English or were accompanied by family members who could translate. After informed consent was given, extensive demographic and epidemiologic data were obtained via a standardized interview conducted on the Qualtrics application (Qualtrics, Provo, UT) for iPad. The Qualtrics survey application provided a high level of quality control and standardization across sites and interviewers, and the offline mode ensured that data were captured and stored securely even when Internet access was inconsistent. The questionnaire interview lasted an average of 45 minutes. To calibrate blood glucose measures, patients were asked about time since their last meal, because it was not feasible to inform patients to fast overnight before recruitment.

Next, the research nurse obtained physical measurements, including height (Seca 217 Portable Stable Stadiometer; Seca, Hamburg, Germany), weight (Seca Flat Scale Model 876), waist circumference (Lufkin W606 pm measuring tape), and blood pressure (average of two measurements; Omron HEM-907XL; Omron, Kyoto, Japan) using equipment standardized across all study sites. In addition, two 8-mL venous blood samples were obtained (BD Vacutainer Safety-Lok blood collection set; BD, Franklin Lakes, NJ), and stored in purple top (BD K2 EDTA; Thermo Fisher Scientific, Waltham, MA) and plain vacutainer blood collection tubes, and saliva samples were stored directly in an Oragene Saliva Collection Kit (ORG 500; DNA Genotek, Ottawa, Canada). After data and sample collection, a routine biopsy was performed. Sterile tissue containers (PAXgene Dual Chamber Tissue Container and 2-mL cryovial; QIAGEN, Hilden, Germany), a biopsy needle (Temno T149 14G Semi-Automatic Biopsy needle, Merit Medical, South Jordan, UT), and ancillary materials (eg, surgical gloves, methylated spirit, bandages, and lidocaine) were provided by the study for routine pathologic evaluation, as well as tumor and adjacent normal tissue collection (from the affected breast) for research purposes. Finally, the patient was provided with an incentive of a N500 (equivalent to approximately US$ 1.50) telephone recharge card for their participation, and the biopsy needle (N14,000 locally; approximately US$ 53) was provided by the study for free (patients are normally required to purchase the needle and other ancillary items needed for the biopsy from personal funds). The research samples were immediately (within 30 minutes) delivered to the laboratory technician for processing and storage in −80°C freezers. Routine treatment and follow-up continued for patients as usual.

### Sample Handling and Shipment

Biologic specimens were labeled with unique patient identifying numbers (PIDs), data of collection and sample type, and then stored in −80°C freezers in institutional laboratories. Laboratory freezers are powered year-round using a combination of national electricity supply, back-up power generators, and inverters. Every 3 to 4 months, samples were packaged in freezer boxes, arranged in dry shippers charged with liquid nitrogen (a method validated to remain at approximately −150°F for up to 14 days), and shipped to the United States. Liquid nitrogen was available for purchase in Lagos (BOC Gases, Lagos), and dry shippers were initially charged, transported back to the laboratory to be stocked and recharged, and then picked up by a World Courier representative (World Courier Inc). The dry shipper with samples was delivered to the Pathology Shared Resource Facility at the University of Kentucky Markey Cancer Center within 5 to 7 days, and samples were immediately transferred into −80°C freezers.

### Data Handling, Management, and Processing

Research nurses at each institution maintained a patient-tracking database that was continuously updated with information on date of recruitment, sample or data collected (ie, interview, blood, tumor, normal, saliva), incentive received, contact information, next of kin, and notes on recruitment process. De-identified recruitment and clinical data from each site, labeled with a unique PID, were sent to the MEND data manager in the United States for integration into a single data management system. Survey data from Qualtrics were downloaded and checked for completeness and consistency. A US pathologist reviewed each tissue sample for pathologic verification of tumor status and immunohistochemistry (IHC) staining for receptors of estrogen, progesterone, human epidermal growth receptor 2, androgen, and Ki-67 was performed. Blood samples were analyzed at a clinical research laboratory to assess for cholesterol, triglyceride, and blood glucose levels. In addition, pathologically verified tumor and adjacent normal samples were sent to a genomics laboratory for epigenome-wide DNA methylation analysis. Survey and clinical data, as well as results from the pathology, biomarker, and methylation assays, were cleaned and integrated into the final study analytical data set. The MEND data manger conducted detailed quality checks on all data items and requested clarification from local interviewers and research coordinators for missing or inconsistent variables when needed. All corrections were carefully annotated and integrated into the main study database for additional analysis. Each institution received a data set with data on patients recruited at their institutions only identified with the PID (to be decoded by site research nurses) as well as laboratory results from US pathology, IHC, and clinical assays for comparative purposes.

### Personnel Training and Quality Control

Research nurses were carefully selected by site collaborators and trained extensively to ensure that study protocols were followed closely, errors in data collection were minimized, and biologic samples were handled safely and properly to ensure quality. Research nurses were trained to emphasize the importance of patient privacy and confidentiality, and were monitored for sensitivity to patients who were receiving a new cancer diagnosis—a devastating experience in any environment, but especially in Nigeria, where cancer stigma still exists. Research nurses completed human subjects’ protection and extensive patient-interview training before working on the project.

### Healthy Controls

Rather than recruit controls de novo as previously described, healthy controls were selected from a cohort of 4,000 women recruited as part of the Human Heredity and Health (H3) Africa Chronic Kidney Disease (CKD) Case-Control Study in Nigeria, predominantly from Lagos, Ogun, Oyo, and Osun states (the same geographic region as the MEND study), and age matched (± 6 months) with breast cancer cases at enrollment. Sample and data collection protocols were similar between the CKD and MEND studies. Specifically, each breast cancer case was matched on age with one healthy control woman without replacement, so that once a control was matched, she was ineligible for matching with other cases. A nearest-matching approach was used such that if there was no control with age ± 6 months of the case, then the control closest in age was chosen. This approach has been used routinely in matched case-control studies.^36-38^ Important study covariates such as sociodemographics, physical measurements, reproductive factors, and family and personal health for the CKD controls were obtained, as well as 1 mL of serum for biomarker analysis of MetS components.

### Participant Follow-Up

Active and passive surveillance of recruited patients was conducted to ascertain fatal and nonfatal cancer outcomes. Patients were contacted by telephone every 12 months around the time of their recruitment anniversary, and additional data were collected on vital status, self-reported physician diagnosis of recurrence, metastasis or remission, adverse effects of treatment, and quality of life. If the patient was unable to respond to follow-up calls or the patient could not be reached, the next of kin was contacted to complete the interview. Working closely with institutional collaborators, research nurses requested access to patient medical records 6 to 12 months after diagnosis and recruitment, and resident doctors assisted with abstracting relevant information on pathology, surgery, chemotherapy, radiation, recurrence, or metastasis.

## RESULTS

### Current Status

A total of 416 patients with breast cancer have been recruited to date (Table 1), 372 of whom have provided blood samples, and 310 also with tumor tissue sample. Approximately 15% of probable breast cancer cases were benign, based on initial pathologic evaluation by Nigerian pathologists. Overall, 15 patients were approached about participation but refused, and one patient initially agreed to participate but eventually withdrew from the study.

**TABLE 1:**
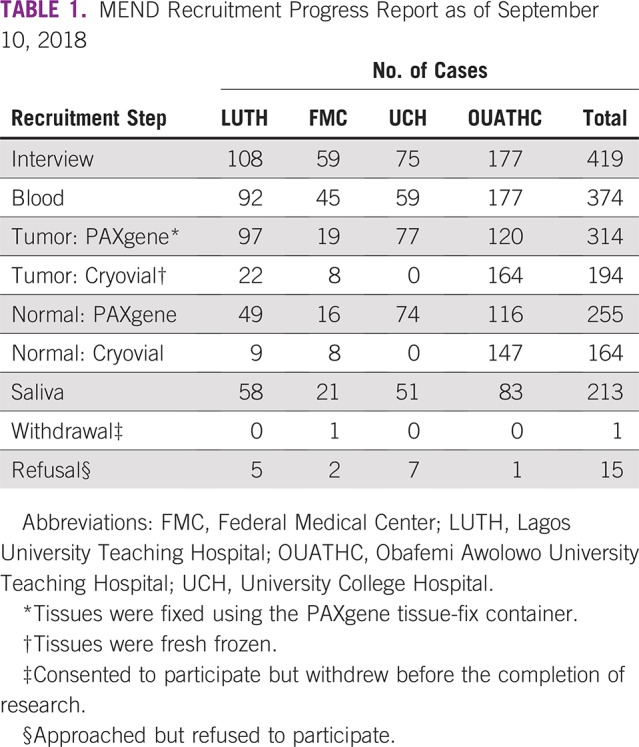
MEND Recruitment Progress Report as of September 10, 2018

As expected, there was no statistically significant difference in age between cases and controls, as a result of matching (Table 2). However, cases compared with controls were slightly older, on average, at menarche (15.43 *v* 15.37 years) and menopause (48.15 *v* 47.88 years), had higher diastolic blood pressure (80.17 *v* 79.13 mm Hg), were less likely to self-report a history of hypertension (17.54% *v* 45.79%) or diabetes (2% *v* 19%), and had slightly fewer pregnancies (4.36 *v* 4.77).

**TABLE 2:**
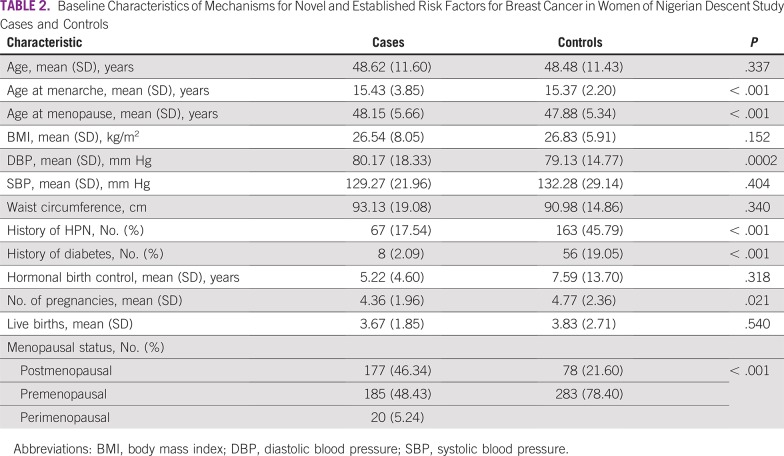
Baseline Characteristics of Mechanisms for Novel and Established Risk Factors for Breast Cancer in Women of Nigerian Descent Study Cases and Controls

## DISCUSSION

The MEND study will generate and integrate patient data at the epigenetic (tissue, blood, and/or saliva samples), molecular (blood and tissue samples), pathologic (IHC and tumor makers), individual (questionnaire data), and clinical (treatment, follow-up) levels to improve understanding of the unique features of breast cancer in Nigeria and to assess the contribution of metabolic dysregulation on breast cancer outcomes. The first MEND scientific papers will focus on the topics highlighted in the introduction—the role of obesity, diabetes, and MetS, and the epigenetic mechanisms linking these exposures with breast cancer incidence and subtypes. We will explore these topics further by examining other novel risk factors for breast cancer in this setting, including metabolic and inflammatory biomarkers, changes in body size over the life course, social determinants, and other genomic markers, and we will re-examine the role of established risk factors for breast cancer subtypes in this setting (eg, reproductive risk factors, diet, physical activity).

The study design for MEND is motivated primarily by the desire to improve understanding and meaningfully affect the prevention and treatment of breast cancer in Nigeria. Although there are several inherent limitations to the case-control study design, such as the potential for differential misclassification of exposures, recall bias, and limitations to external generalizability, this design was the most efficient for the study setting and can serve as a basis for larger prospective studies that may address these limitations.

There are many important scientific questions relevant to improving health and reducing fatal and nonfatal disease outcomes in lower- and middle-income countries, which require better understanding of etiology and biologic mechanisms involved. This information may also enhance treatment strategies in high-income countries. However, there are significant challenges associated with developing high-quality molecular epidemiology studies in resource-limited settings. These may include logistical challenges, difficulty identifying and cultivating scientific collaborations, cultural and language barriers, cost, time frame, and administrative hurdles. Important scientific questions are likely to remain unanswered if these challenges or barriers are not addressed, and, as the MEND study shows, the challenges are surmountable.

Studies using the extensive MEND data and infrastructure will focus on characterizing other genomic features of Nigerian patients with breast cancer and incorporate a prospective assessment of survival. MEND data will foster collaborative studies to better understand breast cancer risk patterns among Africans (Nigerian) and women of the African diaspora, including women of African descent in the United States, United Kingdom, and the Caribbean. Given their shared genetic ancestry, these studies may help elucidate key genetic risk factors that may be conserved across generations, and the impact of distinct environmental and/or lifestyle conditions on breast cancer risk. Findings from these studies can enhance the genetic diversity of pooled studies, provide empirical data that can be used to tailor breast cancer prevention strategies to specific risk factors relevant for different racial groups, and reduce the burden of breast cancer globally.

## Figures and Tables

**TABLE A1:**
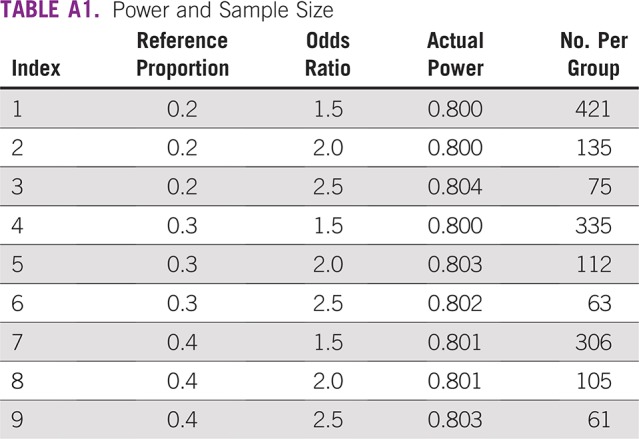
Power and Sample Size

## Appendix

### Analytical Approach

Descriptive statistics for baseline sociodemographic, clinical factors and metabolic syndrome (MetS) components will be conducted to assess for significant differences between cases and controls. Given the matched study design, conditional logistic regression analysis will be used to examine the association between the main exposure variable and the outcome of interest (eg, MetS and breast cancer hormone receptor subtype). All statistical models will be adjusted for potential confounding by age, marital status, socioeconomic status, family history of cancer, and reproductive history. *P* = .05 will be considered statistically significant and for interaction terms, *P* ≤ .1 will be considered significant. All epidemiologic analyses will be conducted using SAS **(**SAS Institute, Cary, NC). For methylation data, we will fit linear mixed-effect regression models with the CpG β score (approximating percent DNA methylation at a CpG site) as the outcome, and MetS (or individual component) and tumor status as predictors, adjusting for confounders and estimated cell proportions and batch effects. We will correct for multiple comparisons using a Bonferroni correction setting α = 1.1 × 10^−7^, and also use false discover rate correction for multiple testing. We will construct Manhattan plots to present results of our epigenome-wide association analysis of MetS, and use mixed models to compare methylation of the top CpG sites by MetS and individual component and tumor status.

### Sample Size and Power

With prevalence of MetS ranging from 20% to 40%, on the basis of prior reports, and setting α at .05, we calculated the minimum sample size needed to maintain 80% power. On the basis of this analysis, we set our minimum sample size at 350 per group (700 total) to detect an odds ratio (OR) of at least 1.50 for the association between MetS and incident breast cancer diagnosis if MetS prevalence is at least 30%. On the basis of the reported prevalence of the least common breast cancer subtype (triple-negative breast cancer [TNBC]) in Nigeria of 32%^39^ and observed OR of the association between MetS and TNBC of 2.40,^40^ we should have the 112 (32% of 350) women with TNBC needed to detect an OR of 2.0 for the association between MetS and TNBC subtype with 80% power. We calculated the minimum sample size required to detect at least a 10% methylation difference at specific CpG sites between tumor and normal tissues, setting power at 80% and α at 10^−5^ to correct for multiple comparisons. We chose 10% difference as a conservative estimate, because prior studies routinely reported two- to three-fold methylation differences between tumor and normal tissues. Because tumor and normal tissues are from the same patient, we anticipate low estimated variance and large tumor-normal differences. On the basis of this analysis, the study is adequately powered to detect at least a 10% methylation difference with a sample size ranging from 13 (if SD = 5) to 40 (if SD = 12).
